# A novel role of glutathione *S*-transferase A3 in inhibiting hepatic stellate cell activation and rat hepatic fibrosis

**DOI:** 10.1186/s12967-019-2027-8

**Published:** 2019-08-23

**Authors:** Haihua Chen, Qixin Gan, Congying Yang, Xiongqun Peng, Jiao Qin, Sisi Qiu, Yanzhi Jiang, Sha Tu, Ying He, Shenglan Li, Huixiang Yang, Lijian Tao, Yu Peng

**Affiliations:** 1 Department of Gastroenterology, Xiangya Hospital, Central South University, 87 Xiangya Road, Changsha, 410008 Hunan China; 2 Department of Radiology, Zhuzhou Hospital of Traditional Chinese Medicine, The First Affiliated Hospital of Hunan College of Traditional Chinese Medicine, Zhuzhou, 412000 China; 3 Department of Endoscopic Medical Center, The Affiliated Cancer Hospital of Xiangya School of Medicine, Central South University, 283 Tongzipo Road, Changsha, 410013 China; 4 Department of Gastroenterology, Changsha Central Hospital, 161 South Shaoshan Road, Changsha, 410004 China; 5 Department of Nephropathy, Changsha Central Hospital, 161 South Shaoshan Road, Changsha, 410004 China; 6 Department of Ultrasonography, The Third Xiangya Hospital, Central South University, 138 Tongzipo Road, Changsha, 410013 China; 7 Department of Nephropathy, Xiangya Hospital, Central South University, 87 Xiangya Road, Changsha, 410008 China

**Keywords:** Glutathione *S*-transferase A3, Hepatic fibrosis, Fluorofenidone, Hepatic stellate cells

## Abstract

**Background and aims:**

Glutathione *S*-transferase A3 (GSTA3) is known as an antioxidative protease, however, the crucial role of GSTA3 in liver fibrosis remains unclear. As a recently we developed water-soluble pyridone agent with antifibrotic features, fluorofenidone (AKF-PD) can attenuate liver fibrosis, present studies were designed to explore the role of GSTA3 in liver fibrosis and its modulation by AKF-PD in vivo and in vitro.

**Methods:**

Rats liver fibrosis models were induced by dimethylnitrosamine (DMN) or carbon tetrachloride (CCl4). The two activated hepatic stellate cells (HSCs) lines, rat CFSC-2G and human LX2 were treated with AKF-PD respectively. The lipid peroxidation byproduct malondialdehyde (MDA) in rat serum was determined by ELISA. The accumulation of reactive oxygen species (ROS) was measured by dichlorodihydrofluorescein fluorescence analysis. The expression of α-smooth muscle actin (α-SMA), fibronectin (FN), and phosphorylation of extracellular signal-regulated kinase1/2 (ERK1/2), p38 mitogen-activated protein kinase (p38 MAPK), c-Jun N-terminal kinase (JNK) and glycogen synthase kinase 3 beta (GSK-3β) were detected by western blotting (WB).

**Results:**

GSTA3 was substantially reduced in the experimental fibrotic livers and transdifferentiated HSCs. AKF-PD alleviated rat hepatic fibrosis and potently inhibited HSCs activation correlated with restoring GSTA3. Moreover, GSTA3 overexpression prevented HSCs activation and fibrogenesis, while GSTA3 knockdown enhanced HSCs activation and fibrogenesis resulted from increasing accumulation of ROS and subsequent amplified MAPK signaling and GSK-3β phosphorylation.

**Conclusions:**

We demonstrated firstly that GSTA3 inhibited HSCs activation and liver fibrosis through suppression of the MAPK and GSK-3β signaling pathways. GSTA3 may represent a promising target for potential therapeutic intervention in liver fibrotic diseases.

## Background

Liver fibrosis is a common process resulting from chronic liver damage caused by different etiologies, and over repair leads to liver cirrhosis, which imposes a substantial economic burden because of the high mortality rate. However, increasing evidence has indicated the reversibility of liver fibrosis and early cirrhosis [1]. Thus, the control of liver fibrosis is the key to avoiding cirrhosis. Extracellular matrix (ECM) is mainly produced by hepatic stellate cells (HSCs), and the activation of HSCs is a key step in liver fibrosis. Generally, HSCs are considered the key target of antifibrotic treatments [2]. However, the mechanisms underlying liver fibrosis and HSC activation remain unclear, and further studies investigating new molecular mechanisms are still needed.

Generally, oxidative stress is a common phenomenon resulting from liver injury that plays a key role in the pathogenesis of liver fibrosis [3]. When oxidative stress is triggered by various harmful stimuli, the accumulation of excess reactive oxygen species (ROS) leads to the lipid peroxidation (LPO) of unsaturated fatty acids in the biological membrane and the generation of byproducts such as 4-hydroxynonenal (4-HNE) and malondialdehyde (MDA), which result in structural and functional damage to the cell [4]. Inhibition of oxidative damage effectively prevents or even reverses the process of liver fibrosis in multiple animal models [5, 6]. HSCs are activated during liver injury caused by various etiologies [2]. Oxidative stress is a common phenomenon resulting from liver injuries. Furthermore, ROS and lipid peroxidation products such as 4-HNE or MDA have been shown to trigger and perpetuate HSC activation via redox-sensitive signaling pathways [7, 8]. For example, the classic mitogen-activated protein kinase (MAPK) signaling pathway has been considered one of the essential pathways involved in HSC activation, and this pathway is redox-sensitive and triggered by several growth factors. Platelet-derived growth factor-BB (PDGF-BB) is well known for its ability to strongly activate HSCs upon binding to specific transmembrane receptors in subjects with liver fibrosis [9]. In addition to the pathways described above, novel pathways have also received attention. The Wnt/β-catenin signaling pathway has emerged as a fundamental growth control pathway that is typically involved in regulating development or the maintenance of most types of stem cells throughout the animal kingdom [10]. However, according to recent studies, this pathway responds to oxidative stress and prevents HSC activation through a specific mechanism during liver fibrosis [11]. GSK-3β is a key kinase in the Wnt/β-catenin pathway. ROS or 4-HNE can trigger GSK-3β inactivation in a manner dependent on ser9 phosphorylation and subsequently activate the downstream signaling cascade to promote HSC activation and proliferation [12]. The detailed mechanisms by which ROS or 4-HNE activate MAPK and Wnt/β-catenin pathways remain unclear and unknown proteins may be involved in this process. Interestingly, as shown in our previous study, the levels of the antioxidant protease glutathione *S*-transferase A3 (GSTA3) is decreased in fibrotic kidneys [13]. GSTA3 belongs to the GST α-class, which converts lipid peroxides to glutathione conjugates Moreover, GSTA3 is major as a cytosolic protein that is expressed at high levels in the liver, kidney and adrenal gland [14, 15]. New advances confirmed that GSTA3 knockout mice exhibit increased oxidative damage and GSTA3 is generally considered to exhibit hydroperoxidase activity in vivo [16]. However, the pathophysiological role of GSTA3 in liver fibrosis has not been studied. Considering the crucial role of oxidative stress in HSC activation and liver fibrosis, we speculate that GSTA3 may play a unique role in liver fibrosis.

Our previous studies confirmed that fluorofenidone [1-(3-fluorophenyl)-5-methyl-2-(1*H*)-pyridone; AKF-PD], which has undergone phase I clinical trials, reduces intracellular ROS accumulation and possesses potent antifibrotic properties because it inhibits HSC activation partially by suppressing MAPK signaling pathways [17–20]. Thus, we speculate that AKF-PD may regulate the levels of certain proteins to mediate oxidative stress and subsequently inhibit HSC activation.

Therefore, the present study was designed to verify whether GSTA3 is involved in liver fibrosis, elucidate the underlying mechanisms and then prove whether AKF-PD reduces liver fibrosis by regulating GSTA3.

## Materials and methods

### Animals and treatment

The animal experimental protocol was performed in accordance with The Institutional Animal Care and Use Committee of Xiangya School of Medicine, Central South University. Male rats weighing between 200 and 220 g from Slac Laboratory Animal (Shanghai, China) were used in our study. All rats were bred and maintained in an air-conditioned animal house with a commercial diet and water available ad libitum, and all rats received humane care according to the university’s guidelines. In this study, the rat models of hepatic fibrosis were induced by dimethylnitrosamine (DMN) [21] and carbon tetrachloride (CCl4) [19], as described previously. Methods for preparing AKF-PD were described in our previous study [19]. For the DMN model, male albino Wistar rats were randomly divided into the following groups: a normal control group, a DMN model group and a DMN + AKF-PD-treated group (n = 10 animals per group). Hepatic fibrosis was induced by administering DMN for 4 weeks. From the beginning of the experiment, the rats in AKF-PD treatment group were administered AKF-PD intragastrically (500 mg/kg/day) once daily for 4 weeks. For the CCl_4_ model, male Sprague–Dawley rats were randomly divided into the following groups: a normal control group, a CCl_4_ model group, and a CCl_4_ + AKF-PD-treated group (n = 10 animals per group). Hepatic fibrosis was induced by administering CCl_4_ for 8 weeks. Beginning at the ninth week, the rats in the AKF-PD treatment group were administered AKF-PD intragastrically (120 mg/kg/day) once daily for 4 weeks. All rats were starved overnight and anesthetized before euthanasia. Blood serum samples and liver tissues were used analyzed.

### Cell culture and transient transfection assay

The immortalized rat and human stellate cell lines CFSC-2G (American Type Culture Collection, Virginia, USA) and LX2 were used in this study. HSCs were cultured in Dulbecco’s Modified Eagle’s Medium from Gibco supplemented with 10% (v/v) fetal bovine serum, 100 U/ml penicillin, and 100 g/ml streptomycin (Invitrogen, California, USA) at 37 °C in a humidified atmosphere of 5% CO_2_ and 95% air. Hepatic stellate cells were transiently transfected with pcDNA3.1(+)-GSTA3 and pcDNA3.1(+) purchased from Genepharma Technology (Shanghai, China). All plasmids were prepared to be endotoxin-free (Qiagen, Valencia, CA). The Silencer Select Predesigned siRNAs targeting GSTA3 were purchased from Thermo Fisher Scientific, Inc. Cells were prepared in 6-well plates. When cells reached 70–80% confluence, plasmids (2 µg per well) or siRNAs (20 nM) were introduced using Lipofectamine 2000 (Invitrogen) according to the manufacturer’s instructions.

### Other materials and methods

Other materials and methods, including reagents, histology procedures using animal tissues, MDA quantification, ROS measurements, quantitative real-time PCR, western blotting, and detailed descriptions of cell treatments, are described in Additional file 1: Materials and methods.

### Statistical analysis

All data are presented as means ± standard deviations. Statistical tests were performed using SPSS 22.0 software (SPSS 22.0, Inc., Chicago, IL, USA). Unpaired Student’s t-tests or one-way ANOVA were performed to analyze the statistical significance of differences between two samples or multiple samples, respectively. Multiple comparison tests were applied only when a significant difference was determined using ANOVA. P < 0.05 was considered statistical significance.

## Results

### GSTA3 expression is decreased in DMN- or CCl_4_-induced liver fibrosis and AKF-PD restores the expression of GSTA3

**Fig. 1:**
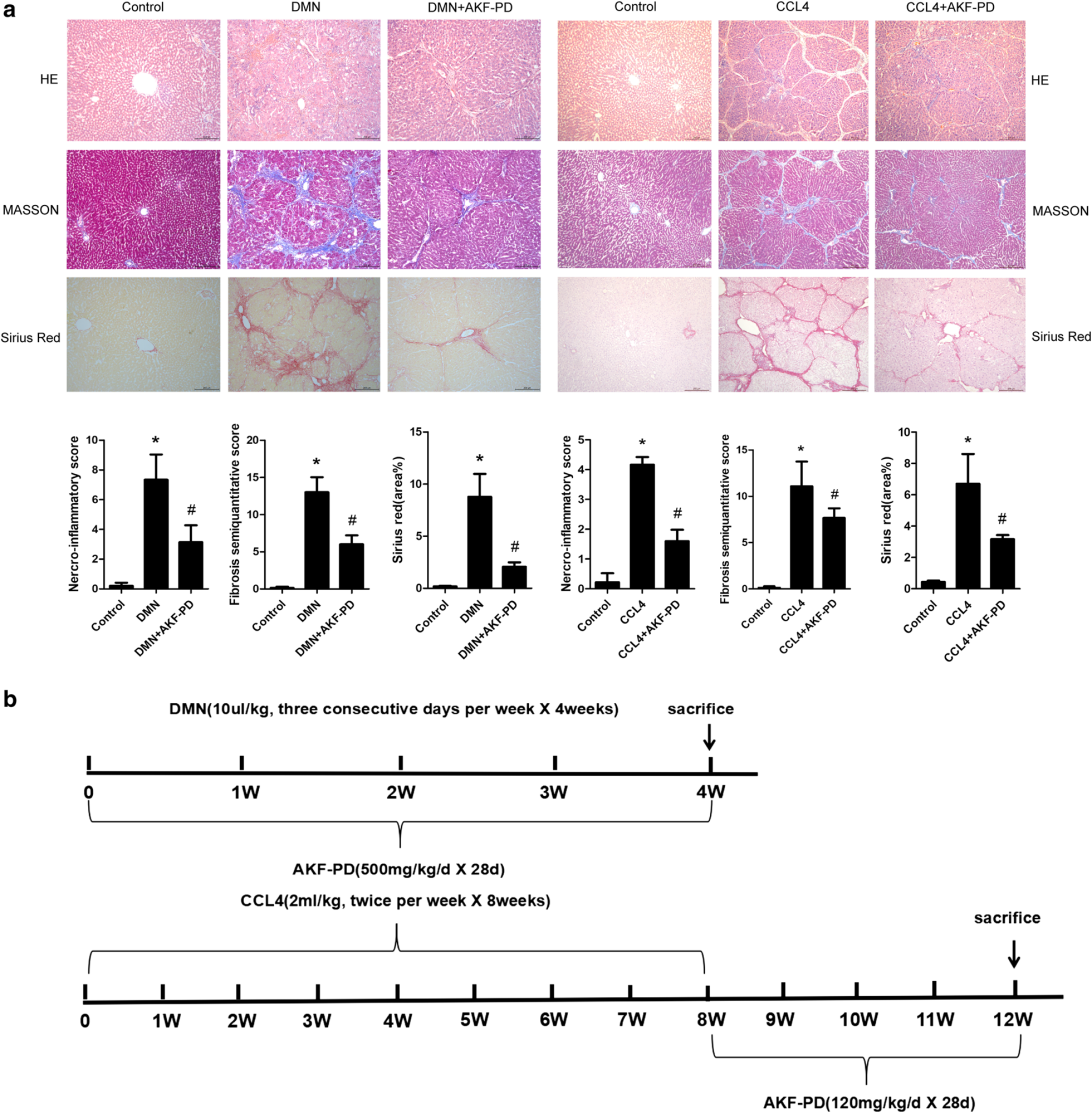

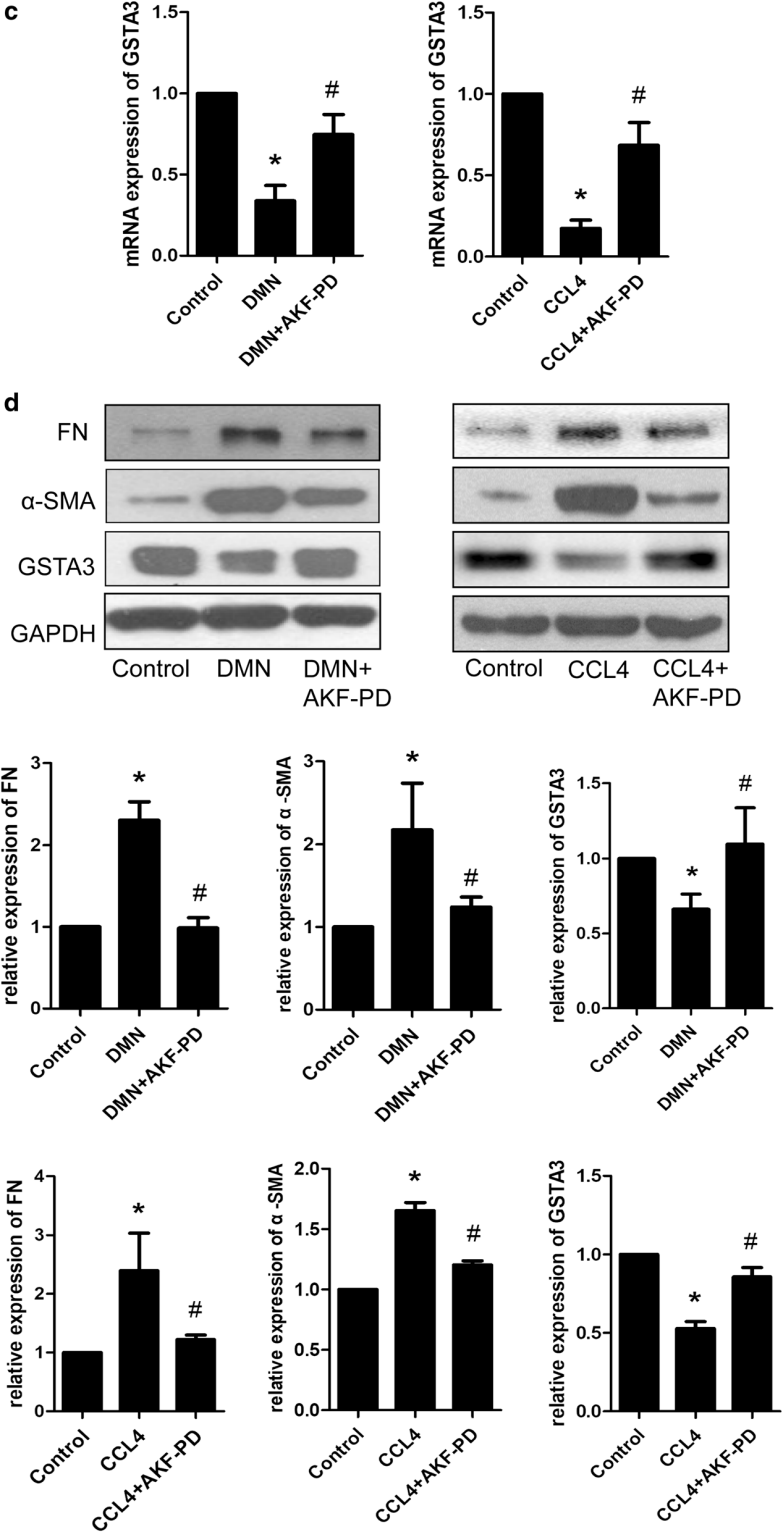

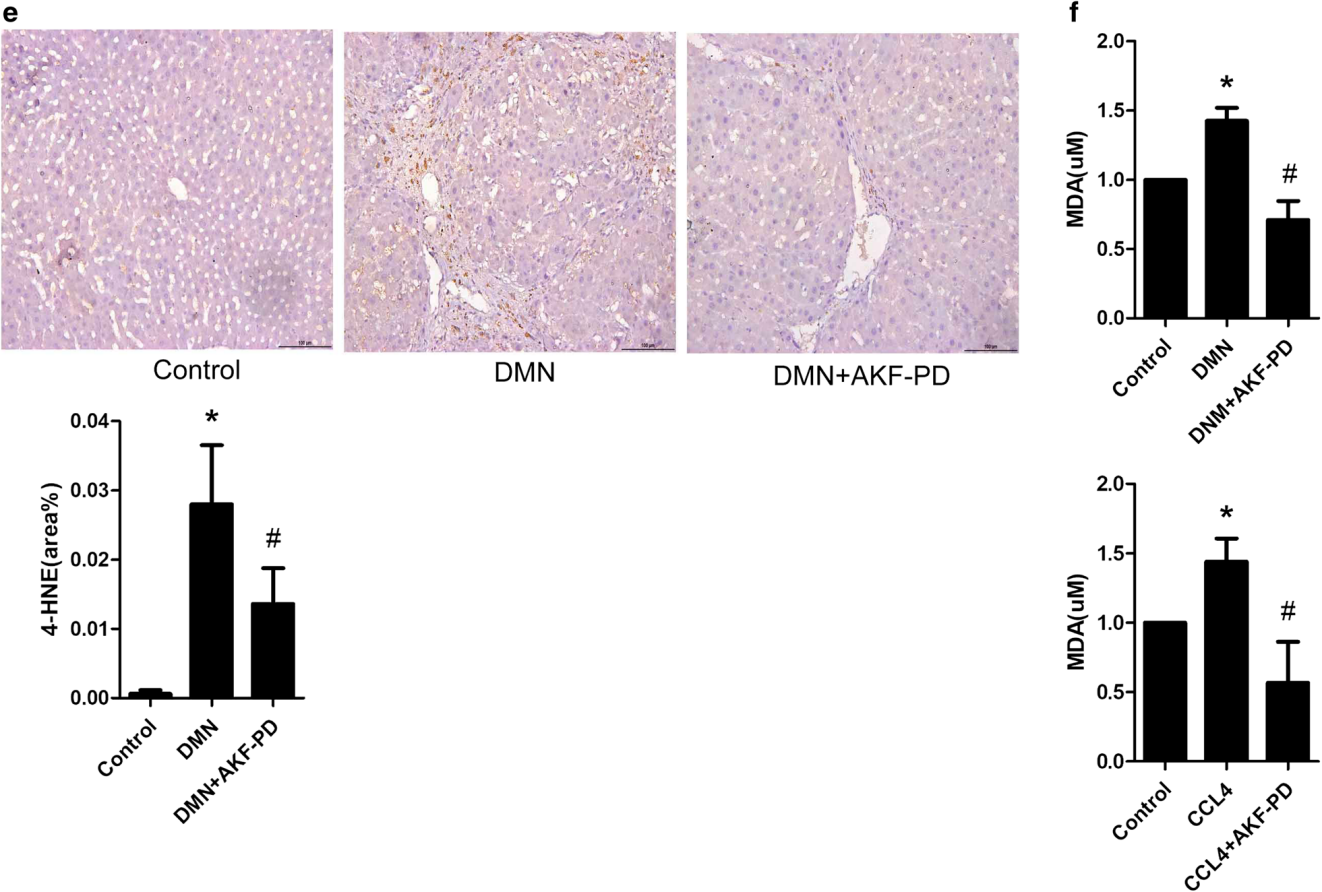
GSTA3 levels were decreased in DMN- and CCL_4_-induced rat liver fibrosis, and AKF-PD restored GSTA3 expression. **a** DMN and CCL_4_ induced fibrosis in the rat liver. Representative photographs from each experimental group are shown (n = 10). Scale bar = 200 µm. **b** Schematic depicting the induction of experimental hepatic fibrosis by DMN or CCl_4_ in rats and the administration of AKF-PD by oral gavage. Hepatic fibrosis was induced by intraperitoneal injections of 10 μl of DMN (diluted 1:100 with 0.15 M NaCl) per kg of body weight or 2 ml of CCl_4_ (diluted 1:1 in olive oil) per kg of body weight. **c** RT-PCR analysis of the hepatic levels of the GSTA3 mRNA (n = 3). **d** WB analysis of the hepatic levels of the GSTA3, α-SMA and FN proteins. GAPDH levels were analyzed as a loading control (n = 3). **e** Immunohistochemical staining for 4-HNE in rat liver sections. (n = 5). Scale bar = 100 µm. **f** ELISA of MDA levels in rat serum (n = 3). Values are presented as mean ± SD. *P < 0.05 compared with the control group and ^**#**^P < 0.05 compared with the DMN or CCl_4_ group. GSTA3, glutathione *S*-transferase A3; DMN, dimethylnitrosamine; CCl_4_, carbon tetrachloride; AKF-PD, fluorofenidone; α-SMA, α-smooth muscle actin; FN, fibronectin; GAPDH,; 4-HNE, 4-hydroxynonenal; MDA, malondialdehyde

Histopathological examinations showed that DMN and CCl_4_ successfully induced hepatic fibrosis in rats, as evidenced by increased collagen fiber accumulation (Fig. 1a). As shown in Fig. 1b, we treated rats with AKF-PD for 4 weeks. Notably, AKF-PD relieved liver fibrosis induced by DMN or CCl_4_ (Fig. 1a). Furthermore, the WB analysis showed significantly increased levels of the α-smooth muscle actin (α-SMA) and fibronectin (FN) proteins in the fibrotic liver compared to normal rat liver tissues, and AKF-PD effectively blocked the expression of the α-SMA and FN proteins in fibrotic livers (Fig. 1d). At the same time, a significant increase in oxidative stress was observed. Immunohistochemical staining revealed a significant increase in the accumulation of the 4-HNE-protein conjugates throughout the fibrotic area following treatment with DMN (Fig. 1e), and substantially higher serum MDA levels were observed in the DMN- and CCl_4_-treated rats than in the normal rats (Fig. 1f). The AKF-PD treatment attenuated the increases in 4-HNE adduct accumulation and MDA levels induced by DMN and CCl_4_ (Fig. 1e, f). Interestingly, levels of the GSTA3 mRNA and protein were significantly decreased in fibrotic rat livers, and AKF-PD dramatically restored both the levels of the GSTA3 mRNA and protein to approximately the levels observed in normal livers (Fig. 1c, d).

### GSTA3 is downregulated in activated HSCs and is upregulated by AKF-PD

**Fig. 2:**
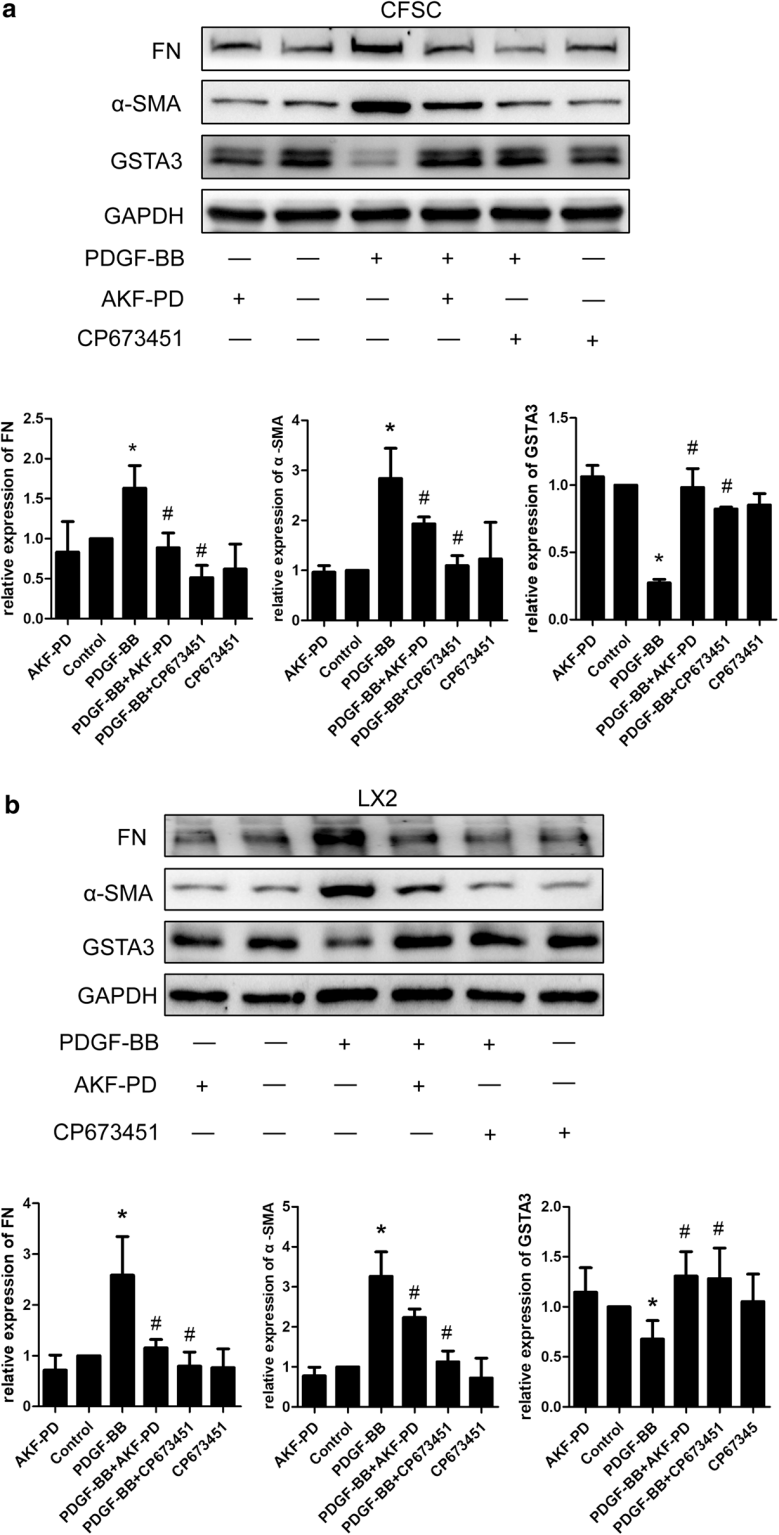

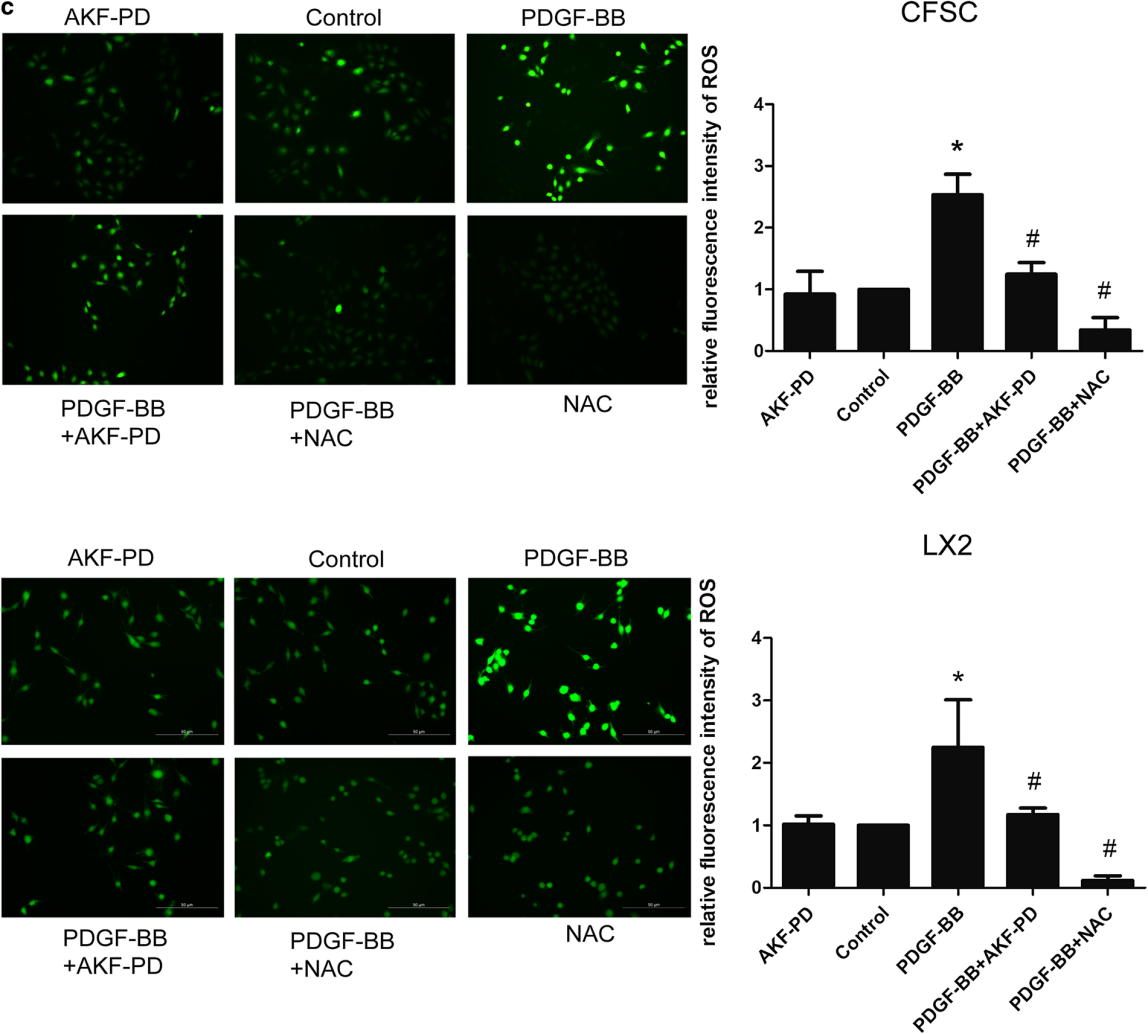
GSTA3 was downregulated in activated HSCs stimulated with PDGF-BB, and AKF-PD upregulated GSTA3 expression. **a**, **b** WB analysis of the levels of the α-SMA, FN and GSTA3 proteins in HSCs. GAPDH levels were analyzed as a loading control. **c** AKF-PD reduced ROS accumulation in HSCs. Fluorescence microscopy images of ROS in HSCs. Scale bar = 50 µm. Flow cytometry was also used to analyze ROS accumulation in HSCs. Values are presented as the mean ± SD of three independent experiments. *P < 0.05 compared with the control group and ^#^P < 0.05 compared with the PDGF-BB treatment group. PDGF-BB, platelet-derived growth factor-BB; ROS, reactive oxygen species; NAC, *N*-acetylcysteine

We detected GSTA3 expression in two HSC cell lines, CFSC-2G and LX2, to confirm the participation of GSTA3 in liver fibrosis. As shown in Fig. 2a, b, the levels of the α-SMA and FN proteins were significantly increased in both CFSC-2G and LX2 cells exposed to PDGF-BB for 24 h compared with the control cells. Surprisingly, the level of the GSTA3 protein was dramatically decreased in activated HSCs induced by PDGF-BB (Fig. 2a, b). Meanwhile, a PDGF-BB receptor inhibitor (CP673451) substantially inhibited HSC activation and abolished the GSTA3 downregulation stimulated by PDGF-BB (Fig. 2a, b). Additionally, the AKF-PD treatment effectively suppressed the PDGF-BB-induced increases in α-SMA and FN levels, and significantly increased the level of the GSTA3 protein in HSCs (Fig. 2a, b). The AKF-PD only control group showed no statistically significant differences in α-SMA, FN and GSTA3 levels compared to the control cells.

Because GSTA3 functions as an antioxidant protease, we evaluated ROS levels in HSCs, and the ROS scavenger N-acetylcysteine served as a negative control. As shown in Fig. 2c, PDGF-BB significantly increased intracellular ROS accumulation in HSCs. Either the AKF-PD or NAC pretreatment significantly eliminated PDGF-BB-induced intracellular ROS accumulation. However, no significant difference was observed between the control group and AKF-PD pretreatment alone.

### GSTA3 inhibits HSC activation and fibrogenesis

**Fig. 3:**
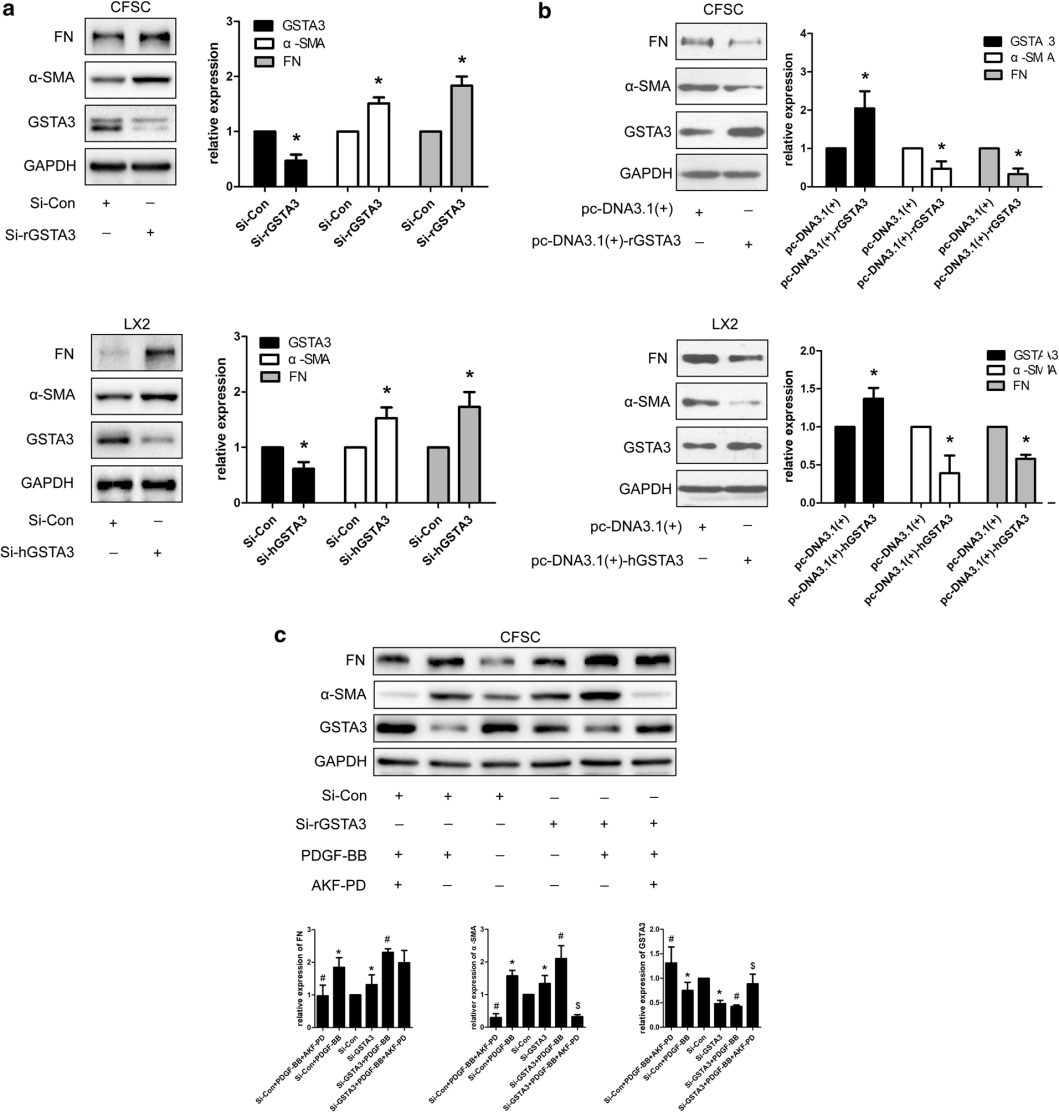

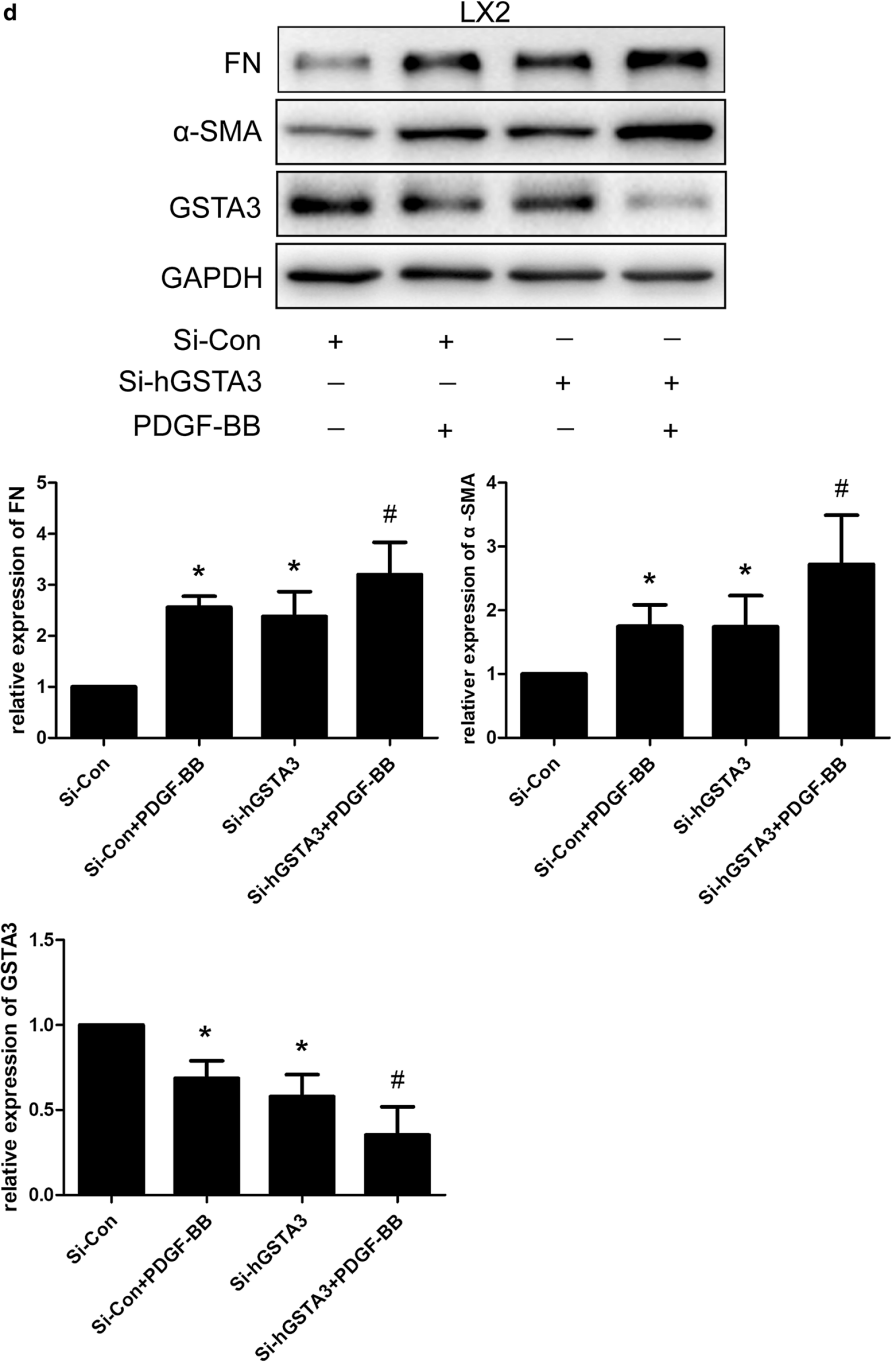

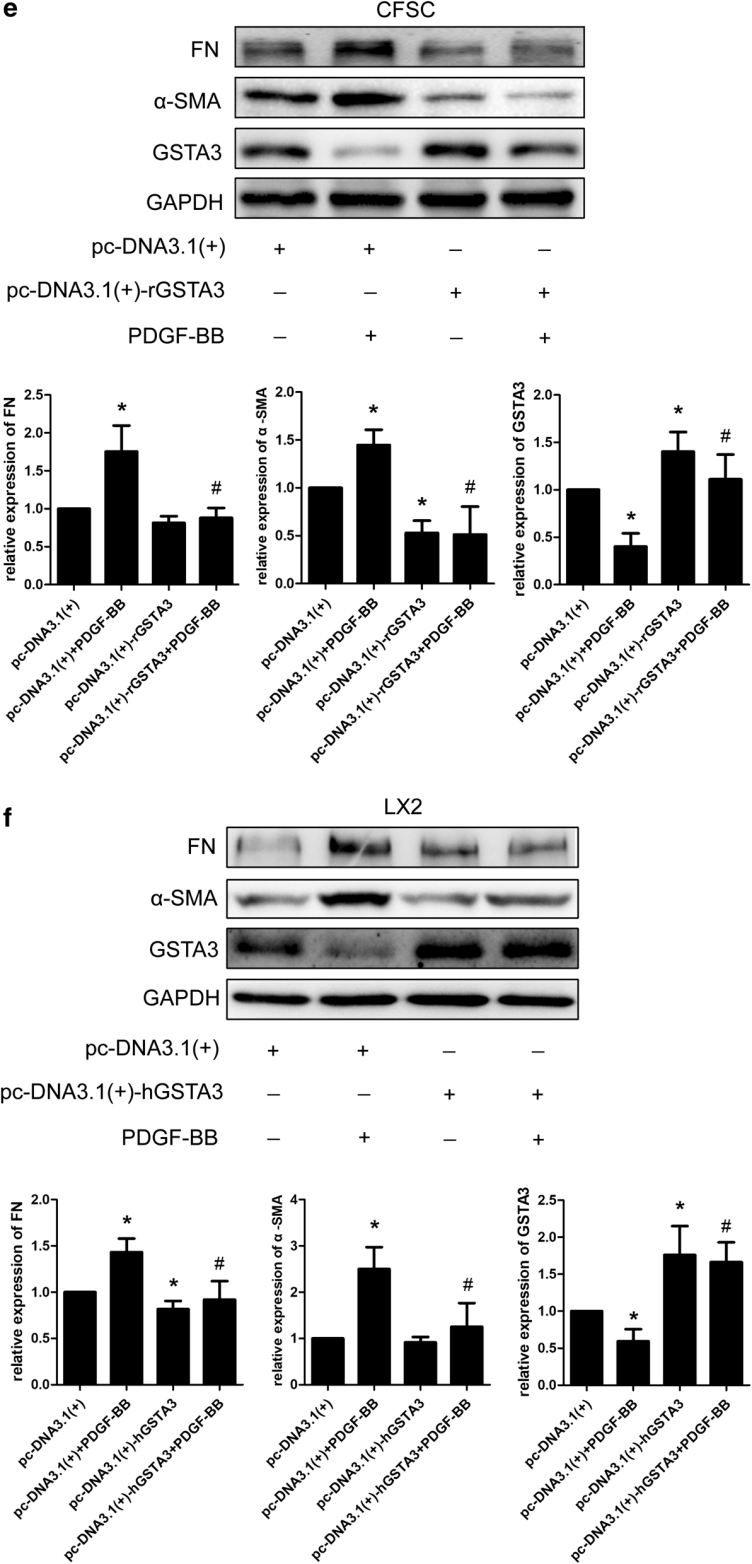
GSTA3 inhibited HSC activation and fibrogenesis. **a** WB analysis of α-SMA and FN levels in CFSC-2G and LX2 cells. After an incubation with the GSTA3 siRNA-Lipofectamine 2000 complex for 6 h, HSCs were maintained in culture medium for 48 h before harvest for the protein analysis. **b** WB analysis of α-SMA and FN levels in CFSC-2G and LX2 cells. After an incubation with the GSTA3 plasmid-Lipofectamine 2000 complex for 6 h, HSCs were maintained in culture medium for 48 h before harvest for the protein analysis. c, **d** WB analysis of GSTA3, α-SMA and FN levels in CFSC-2G and LX2 cells. After an incubation with the GSTA3 siRNA-Lipofectamine 2000 complex for 6 h, HSCs were maintained in culture medium for 24 h and subsequently treated with 10 ng/ml PDGF-BB for another 24 h before harvest for the protein analysis. **e**, **f** WB analysis of GSTA3, α-SMA and FN levels in CFSC-2G and LX2 cells. CFSC cells were incubated with the GSTA3 plasmid-Lipofectamine 2000 complex for 6 h and then maintained in culture medium for 24 h and subsequently treated with 10 ng/ml PDGF-BB for another 24 h before harvest for the protein analysis. LX2 cells were incubated with LV-GSTA3 for 24 h. Afterwards, LX2 were incubated with culture medium for another 24 h and stimulated with 10 ng/ml PDGF-BB for an additional 24 h. GAPDH levels were analyzed as a loading control. All values are presented as the mean ± SD of three independent experiments. *P < 0.05 compared with the Si-Con group, pc-DNA3.1(+) group or LV-Con group; ^#^P < 0.05 compared with the Si-Con + PDGF-BB group or pc-DNA3.1(+)+PDGF-BB group. ^$^P < 0.05 compared with the Si-rGSTA3 + PDGF-BB. Si-Con, negative control siRNA; Si-rGSTA3, rat GSTA3 siRNA; Si-hGSTA3, human GSTA3 siRNA; pc-DNA3.1(+), negative control pc-DNA3.1(+); pc-DNA3.1(+)-rGSTA3, rat pc-DNA3.1(+)-GSTA3; pc-DNA3.1(+)-hGSTA3, human pc-DNA3.1(+)-GSTA3

We knocked down and overexpressed GSTA3 in CFSC-2G and LX2 cells to further decipher the causal relationship between GSTA3 and HSC activation. According to the WB analysis, GSTA3 knockdown remarkably increased α-SMA and FN levels (Fig. 3a). In contrast, GSTA3 overexpression dramatically reversed HSC activation and decreased FN secretion (Fig. 3b). Furthermore, PDGF-BB induced a much greater increase in α-SMA and FN levels after GSTA3 knockdown (Fig. 3c, d). However, AKF-PD treatments reduced expression of α-SMA and FN in both negtive control HSCs and GSTA3 knocdown HSCs. Surprisely, the GSTA3 expression level is fully restored on treatment with AKF-PD after GSTA3 knockdown (Fig. 3c). Meanwhile, GSTA3 overexpression in combination with a PDGF-BB treatment for 24 h did not induce significant increases in the levels of α-SMA and FN (Fig. 3e, f). Based on these results, GSTA3 is one of the factors inducing HSC activation.

### GSTA3 suppresses intracellular ROS accumulation and the activation of the MAPK and GSK-3β pathways in HSCs

**Fig. 4:**
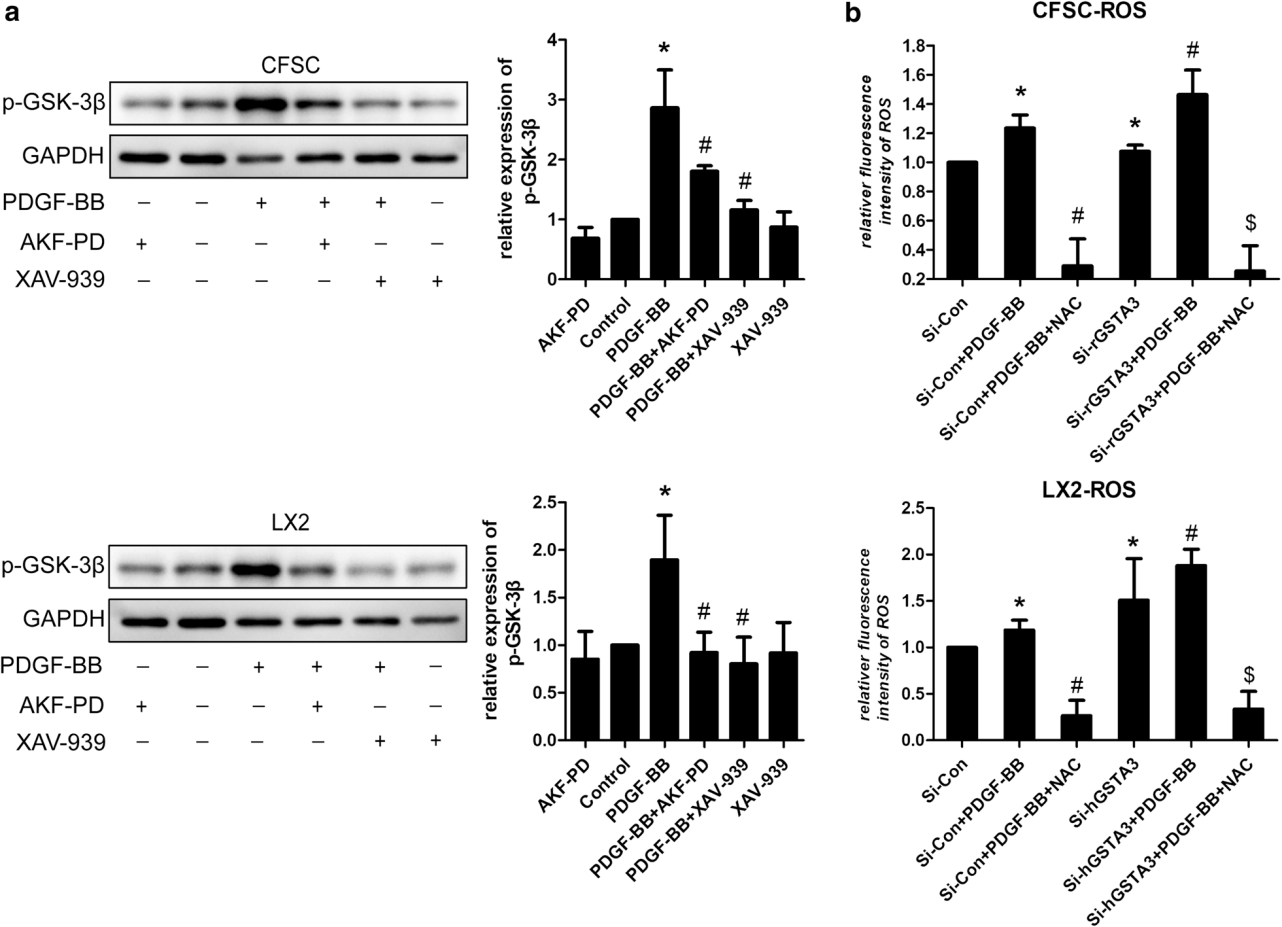

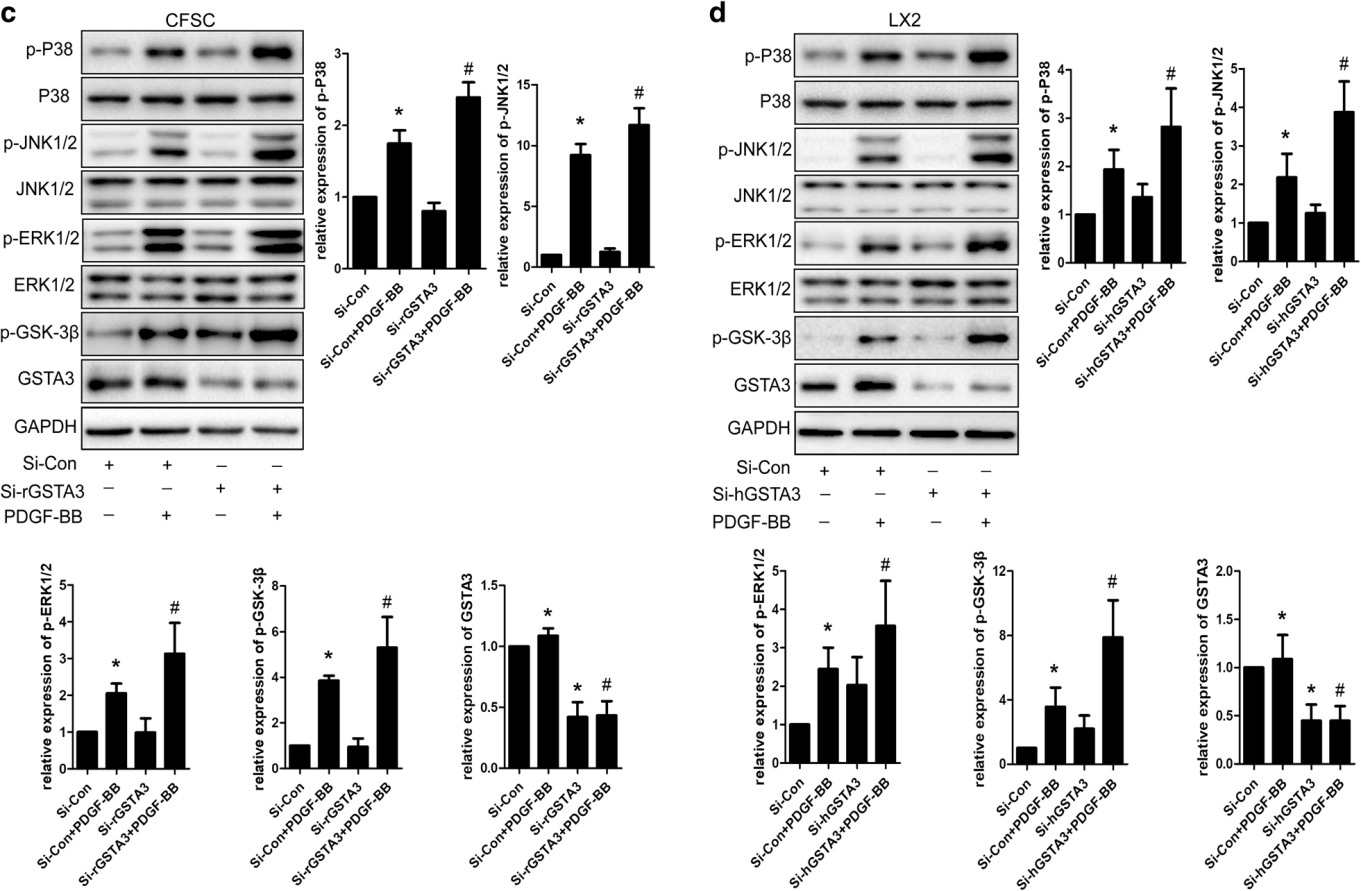

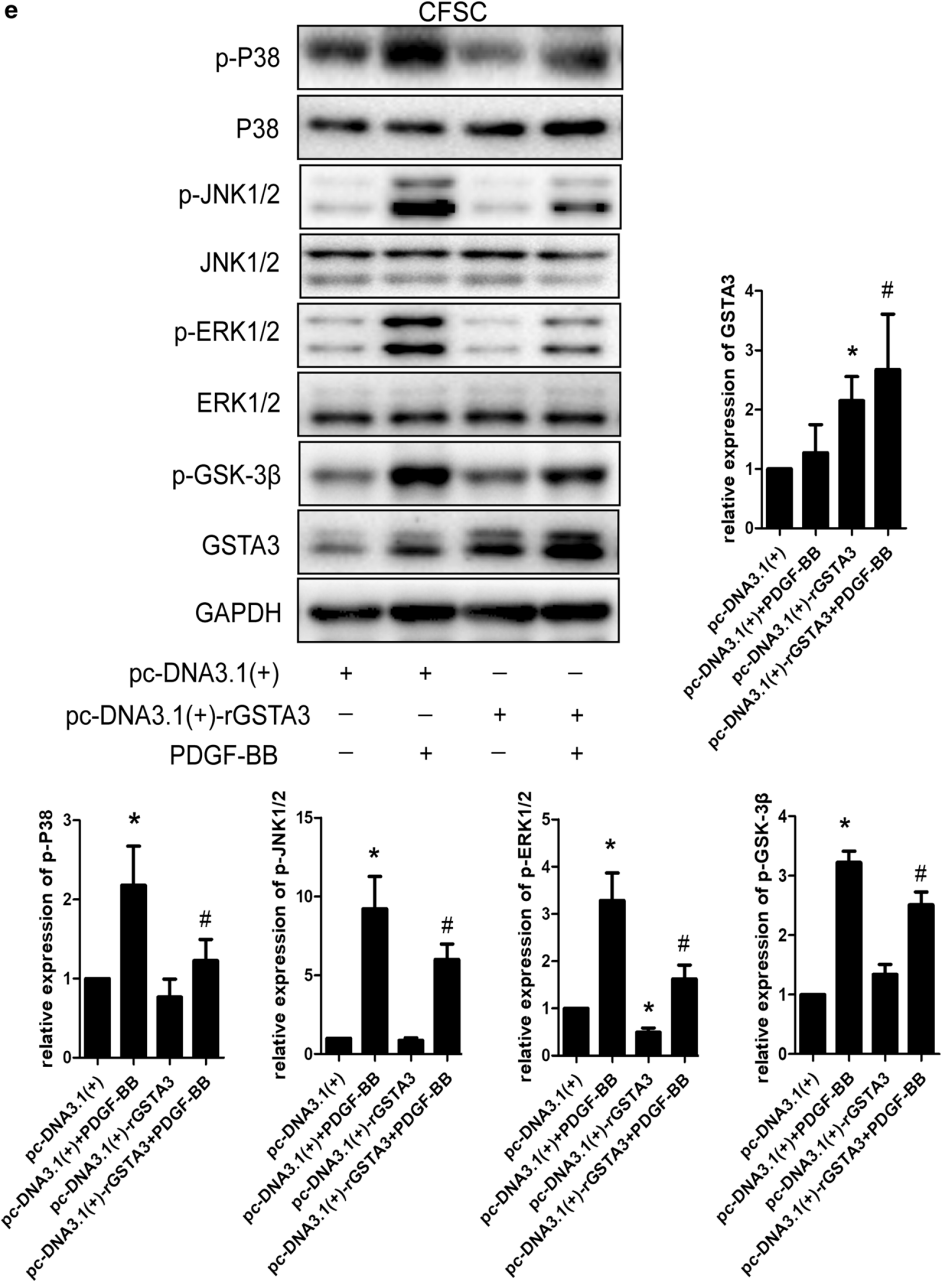
GSTA3 suppressed ROS accumulation and the activation of the MAPK and GSK-3β pathways in HSCs. **a** WB analysis of p-GSK-3β levels. Values are presented as the mean ± SD of three independent experiments. *P < 0.05 compared with the control group and ^#^P < 0.05 compared with the PDGF-BB treatment group. **b** Flow cytometry was used to detect ROS accumulation in HSCs. HSCs were stimulated with PDGF-BB (10 ng/ml) for 30 min after transfection with the GSTA3 siRNA for 48 h. **c**, **d** WB analysis of p-P38, p-ERK, p-JNK and p-GSK-3β levels in CFSC-2G and LX2 cells. After an incubation with the GSTA3 siRNA-Lipofectamine 2000 complex for 6 h, HSCs were maintained in culture medium for 48 h and then stimulated with PDGF-BB (10 ng/ml) for 15 min before harvest for the protein analysis. **e** WB analysis of p-P38, p-ERK, p-JNK and p-GSK-3β levels in CFSC-2G cells. After an incubation with the GSTA3 plasmid-Lipofectamine 2000 complex for 6 h, HSCs were maintained in culture medium for 48 h and then stimulated with PDGF-BB (10 ng/ml) for 15 min before harvest for the protein analysis. The phosphorylation of ERK1/2, P38 and JNK were determined by calculating the ratios to the total ERK1/2, total P38 and total JNK levels, respectively. The level of the p-GSK-3β protein was normalized to GAPDH. Values are presented as the mean ± SD of three independent experiments. *P < 0.05 compared with the Si-Con group or pc-DNA3.1(+) group, ^#^ P < 0.05 compared with the Si-Con + PDGF-BB group or pc-DNA3.1(+)+PDGF-BB group, and ^$^P < 0.05 compared with the Si-rGSTA3 + PDGF-BB or Si-hGSTA3 + PDGF-BB group

We focused on the effects of GSTA3 on intracellular ROS accumulation and the activation of the downstream MAPK and GSK-3β pathways in both CFSC-2G and LX2 cells to elucidate the mechanism by which GSTA3 negatively regulates HSC activation and fibrogenesis. First, we evaluated intracellular ROS levels with the 2′,7′-dichlorofluorescin diacetate probe. PDGF-BB significantly increased ROS generation, and NAC showed an extraordinary ability to clear ROS (Fig. 4b). However, PDGF-BB induced higher levels of ROS production after GSTA3 knockdown (Fig. 4b). Furthermore, PDGF-BB effectively phosphorylated p38 mitogen-activated protein kinase (p38 MAPK), c-Jun N-terminal kinase (JNK), extracellular signal-regulated kinase 1/2 (ERK1/2) and GSK-3β in HSCs (Fig. 4c, d). More importantly, the PDGF-BB treatment induced much higher levels of p-P38, p-JNK, p-ERK1/2 and p-GSK-3β after GSTA3 knockdown in HSCs (Fig. 4c, d). Conversely, overexpression of GSTA3 significantly reduced the levels of p-P38, p-JNK, p-ERK1/2 and p-GSK-3β in cells stimulated with PDGF-BB (Fig. 4e).

Furthermore, we also assessed the effect of AKF-PD on the GSK-3β pathway. As shown in Fig. 4a, a PDGF-BB treatment for 15 min dramatically stimulated GSK-3β phosphorylation in HSCs. Pretreatments with AKF-PD and the GSK-3β inhibitor XAV-939 effectively inhibited GSK-3β phosphorylation, but significant differences were not observed between cells treated with XAV-939 and AKF-PD alone (Fig. 4a).

### AKF-PD attenuates the activation of the MAPK and GSK-3β pathways in vivo

**Fig. 5:**
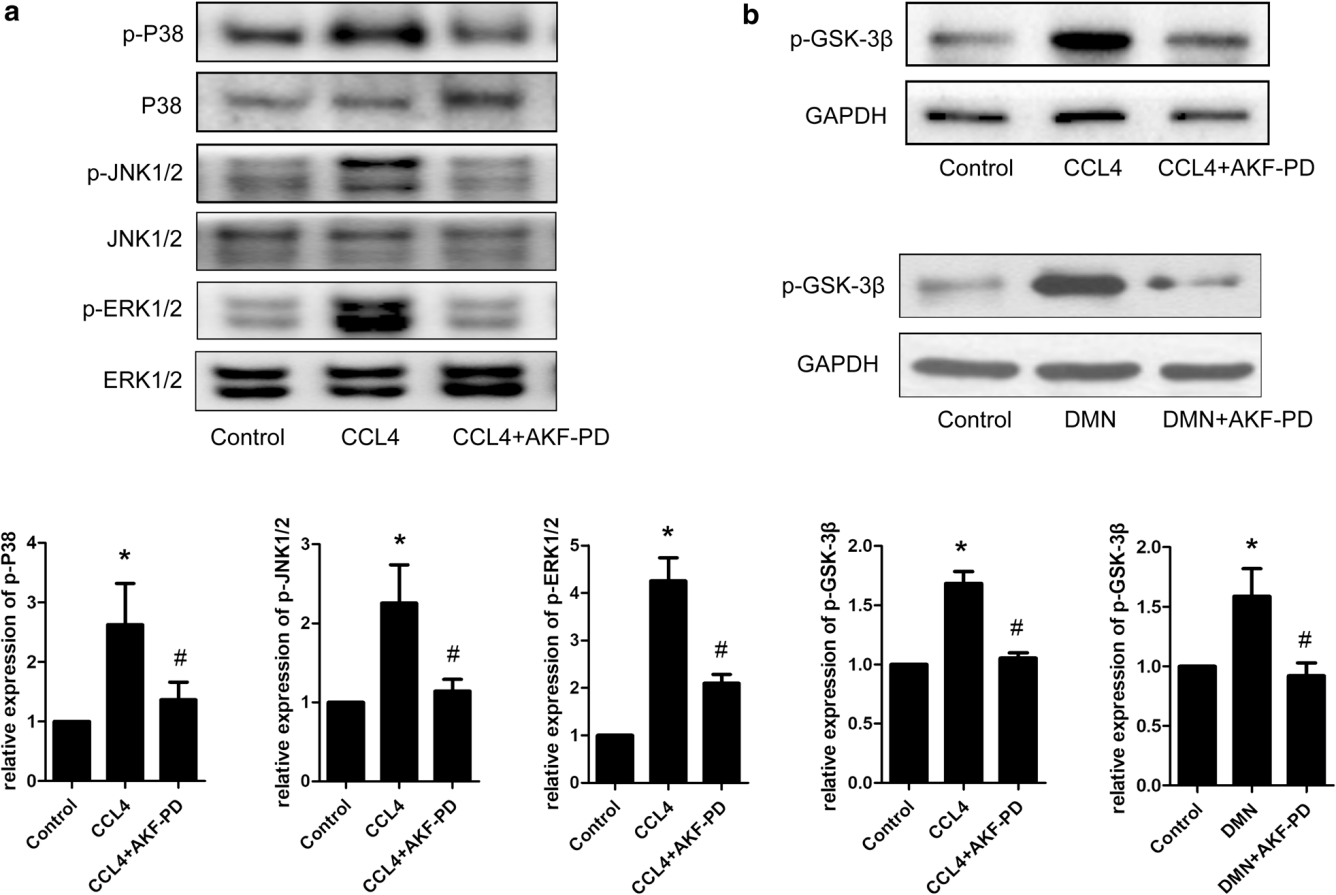
AKF-PD inhibited the MAPK and GSK-3β pathways in vivo. **a** WB analysis of MAPK signaling in rat livers. The p-ERK1/2, p-P38 and p-JNK levels were measured by calculating ratios to the total ERK1/2, total P38 and total JNK levels, respectively. **b** WB analysis of p-GSK-3β levels in rat livers. GAPDH levels were analyzed as a loading control. n = 3. *P < 0.05 compared with the control group and ^#^P < 0.05 compared with the CCl_4_ or DMN group

As shown in our previous studies, AKF-PD relieves liver fibrosis and suppresses HSC activation by inhibiting MAPK signaling. In vivo, we confirmed that AKF-PD suppressed MAPK signaling induced by DMN in rats [19]. In the current study, CCl_4_ increased p-P38, p-ERK1/2, and p-JNK levels in fibrotic livers, and the AKF-PD treatment successfully decreased the phosphorylation of P38, ERK1/2 and JNK (Fig. 5a). Consistent with the in vitro experiments, a WB analysis showed much higher p-GSK-3β levels in CCl_4_- or DMN-induced fibrotic livers, and the AKF-PD treatment effectively decreased p-GSK-3β levels (Fig. 5b). In summary, AKF-PD attenuated the activation of the MAPK and GSK-3β pathways in vivo.

## Discussion

Liver fibrosis is a common pathological process leading to cirrhosis and eventual liver failure [22]. However, the pathogenesis of liver fibrosis is complicated, and the mechanism remains unclear. In fact, regardless of whether the cause is viral infection, fat- or alcohol-related liver disease, cholestasis, or a metabolic or toxic etiology, HSC activation is a common event that leads to ECM remodeling after evolving to chronic injury [2].

Oxidative stress represents a common link among different etiologies of persistent liver injury. This injury occurs when oxidative stress-related molecules, including ROS and end-products of LPO, exceed the cellular antioxidant defense capabilities. MDA and 4-HNE are important end-products of LPO, and their concentrations reflect the level of LPO [23]. GST family members are important components of the cellular antioxidant defenses and serve as antioxidant enzymes that scavenge end-products of LPO. GSTA3 is a special member of the α-GST subfamily. The antioxidant response elements in the proximal promoter region of the GSTA3 gene are involved in its antioxidant activity [24]. Indeed, oxidative damage is aggravated in GSTA3 knockout mice following exposure to CCl_4_ [16]. In the present study, the expression of GSTA3 was dramatically decreased and the level of oxidative stress increased in rat fibrotic livers and activated HSCs. Only one previous report described the phenomenon in which GSTA3 was decreased and LPO products were significantly increased in rat HSCs [25]. Another report clarified that GSTA3 reduced 4-HNE levels in hepatocytes and regulated the signaling pathways that protect against oxidative stress [26]. Most likely, GSTA3 expression was decreased, resulting in a redox imbalance that lead to excess ROS and LPO accumulation. Both ROS and products of LPO initiate and perpetuate HSC activation [27–29]. Thus, GSTA3 is involved in HSC activation and liver fibrosis by regulating oxidative stress.

PDGF-BB was reported to trigger HSC activation in a manner dependent on ROS generation via NADPH oxidase [30, 31]. PDGF-BB induced ROS production in HSCs in the present study, consistent with the previous report. Since an antioxidant response element is located in the proximal promoter region of the rat and human GSTA3, GSTA3 expression may be triggered by ROS. In contrast, PDGF-BB downregulated GSTA3 expression in HSCs. Thus, we speculate that the expression of the GSTA3 gene is likely regulated by PDGF-BB through a specific mechanism. Of course, further studies are needed to identify the mechanism.

HSCs can transdifferentiate into a myofibroblast-like phenotype and acquire a fibrogenesis capacity. The process of HSC activation consists two major phases: initiation and perpetuation [2]. This process was substantially enhanced by GSTA3 knockdown, and the effect was even more obvious after an incubation with PDGF-BB. Correspondingly, overexpression of GSTA3 inhibited HSC activation and FN production, even after PDGF-BB treatments. Notably, recent advances have identified GSTA3 as a novel adipocyte differentiation-associated protein [32]. However, no investigation has indicated a causal relationship between GSTA3 and HSC differentiation. Our results are the first to show that GSTA3 negatively regulates HSC activation and fibrogenesis. This study unequivocally identified GSTA3 as one factor inducing HSC activation.

Various etiologies leading into liver fibrosis drive the differentiation of HSCs into a myofibroblast-like phenotype through various signaling networks. MAPK signaling is among the best characterized pathways involved in the HSC phenotype switch, and this pathway is activated by both ROS and 4-HNE [33]. On the other hand, ROS and LPO also activate Wnt signaling, which has been reported to play a specific role in HSC activation and proliferation in hepatic fibrosis. When the canonical Wnt signaling pathway is activated, Wnt proteins bind to the transmembrane receptor complex and inhibit GSK-3β and its detachment from the scaffold protein; this process promotes further β-catenin accumulation in the cytoplasm and transport to the nucleus, thereby initiating the expression of the downstream target genes c-myc, c-Jun, cyclin D1, which mediate cell differentiation and proliferation [10]. Our previous study confirmed that the cyclin D1 participates in the mechanism regulating HSC proliferation [21]. However, PDGF-BB did not significantly induce the transport of β-catenin from the cytoplasm to the nucleus (Additional file 2: Figure S1). Although PDGF-BB is the strongest mitogen in HSCs, it is not a canonical Wnt signaling ligand. Actually, only a very small portion of cellular GSK3 and β-catenin are involved in the Wnt/β-catanin pathway [34]. GSK3 actually participates in multiple signaling pathways. In HSCs, GSK-3β is constitutively active and maintains the HSCs in quiescent state [11]. In the present study, GSTA3 inhibited the inactivation of GSK-3β. Thus, GSTA3 suppressed GSK-3β-mediated activation of HSCs. On the other hand, GSTA3 reduced ROS accumulation and inhibited MAPK signaling in HSCs. The MAPK signaling pathway is critical for liver fibrosis [35]. Collectively, GSTA3 suppressed HSC activation by regulating the activity of the MAPK and GSK-3β signaling pathways, and partially through the negative regulation of oxidative stress in HSCs. Since both the MAPK and GSK-3β signaling pathways are critical for HSC activation, the significant effects of GSTA3 on these pathways are sufficient to clarify its unique role in the activation of HSCs. Although GSTA3 affected intracellular ROS accumulation and its common downstream pathways, we were unable to exclude the possibility that GSTA3 may affect intracellular signal transduction mediated by other pathways. In particular, according to recent evidence, several other GSTs regulate cell signaling pathways through direct protein–protein interactions [36]. Of course, further studies are needed to clarify the mechanisms.

An effective treatment targeting the cause of liver fibrosis reduced and even reversed liver fibrosis in previous studies. Antiviral therapy targeting viral hepatitis has been reported to reverse cirrhosis in some patients [37]. However, the development of an optimal antifibrotic treatment for numerous nonviral liver diseases is more difficult to achieve. Hence, an effective antifibrotic drug might ameliorate the symptoms of these patients. To date, no antifibrotic drug has been approved for liver fibrosis. Approaches designed to attenuate oxidative damage as therapeutic strategies for liver fibrosis are still being investigated [38]. Our previous studies have shown the potent ability of AKF-PD to ameliorate fibrosis in multiple organs [18–21]. Furthermore, AKF-PD inhibits intracellular ROS accumulation, suggesting that AKF-PD possesses antioxidant activity [18]. In the present study, AKF-PD reduced ROS accumulation in HSCs and decreased LPO levels in vivo. Based on the important role of oxidative stress in liver fibrosis, we searched for a key target protein responsible for the antioxidant activity of AKF-PD. GSTA3 is an inducible antioxidant enzyme [39, 40]. Surprisingly, AKF-PD effectively increased GSTA3 expression in fibrotic livers and activated HSCs. Even after GSTA3 knockdown in HSCs, AKF-PD fully restored expression of GSTA3 and effectively inhibited HSCs activation. Obviously, powerful up-regulation of AKF-PD in expression of GSTA3 offsets the effects of GSTA3 knockdown on HSCs. In a word, GSTA3 is a special target of AKF-PD and is at least partially responsible for its anti-fibrotic ability. Further mechanistic studies of the downstream signaling pathways of ROS showed that AKF-PD suppressed the activation of the MAPK and GSK-3β signaling pathways. Strategies targeting the MAPK and GSK-3β signaling pathways exert anti-fibrotic effects on animal models of fibrosis [41, 42]. Collectively, AKF-PD relieved liver fibrosis partially by upregulating GSTA3 expression and negatively regulating oxidative stress and its downstream pathways.

## Conclusions

Our investigation revealed that GSTA3 has unexpected new functions in the inhibition of HSC activation and hepatic fibrosis for the first time. Furthermore, we achieved effective pharmacological regulation of GSTA3 as a target of AKF-PD. In the future, GSTA3 may be developed as a potent therapeutic target for liver fibrosis.

## Supplementary information

**Additional file 1.** Additional materials and methods.

**Additional file 2: Figure S1.** Expression of β-catenin in cytoplasm and nuclear of HSCs.

## Data Availability

All data generated or analyzed during this study are included in this published article.

## Abbreviations

AKF-PD: fluorofenidone
α-SMA: α-smooth muscle actin
CCl_4_: carbon tetrachloride
DMN: dimethylnitrosamine
ECM: extracellular matrix
ERK1/2: extracellular signal-regulated kinase
4-HNE: 4-hydroxynonenal
FN: fibronectin
GSTA3: glutathione *S*-transferase A3
GSK-3β: glycogen synthase kinase-3 beta
HSCs: hepatic stellate cells
JNK: c-Jun N-terminal kinase
LPO: lipid peroxidation
MDA: malondialdehyde
PDGF-BB: platelet-derived growth factor-BB
p38 MAPK: p38 mitogen-activated protein kinase
ROS: reactive oxygen species
WB: western blotting
