# Correction to: A novel role of glutathione *S*-transferase A3 in inhibiting hepatic stellate cell activation and rat hepatic fibrosis

**DOI:** 10.1186/s12967-020-02346-4

**Published:** 2020-04-30

**Authors:** Haihua Chen, Qixin Gan, Congying Yang, Xiongqun Peng, Jiao Qin, Sisi Qiu, Yanzhi Jiang, Sha Tu, Ying He, Shenglan Li, Huixiang Yang, Lijian Tao, Yu Peng

**Affiliations:** 1 Department of Gastroenterology, Xiangya Hospital, Central South University, 87 Xiangya Road, Changsha, 410008 Hunan China; 2 Department of Radiology, Zhuzhou Hospital of Traditional Chinese Medicine, The First Affiliated Hospital of Hunan College of Traditional Chinese Medicine, Zhuzhou, 412000 China; 3 Department of Endoscopic Medical Center, The Affiliated Cancer Hospital of Xiangya School of Medicine, Central South University, 283 Tongzipo Road, Changsha, 410013 China; 4 Department of Gastroenterology, Changsha Central Hospital, 161 South Shaoshan Road, Changsha, 410004 China; 5 Department of Nephropathy, Changsha Central Hospital, 161 South Shaoshan Road, Changsha, 410004 China; 6 Department of Ultrasonography, The Third Xiangya Hospital, Central South University, 138 Tongzipo Road, Changsha, 410013 China; 7 Department of Nephropathy, Xiangya Hospital, Central South University, 87 Xiangya Road, Changsha, 410008 China

## Correction to: J Transl Med (2019) 17:280 10.1186/s12967-019-2027-8

Following publication of the original article [1], the authors identified an error in the corresponding authorship. In the original article, Yu Peng is indicated as the sole corresponding author. However, Yu Peng shares corresponding authorship with Huixiang Yang.

The corrected corresponding authorship is reflected in this correction.
