# Supplementary material for: A novel role of glutathione S-transferase A3 in inhibiting hepatic stellate cell activation and rat hepatic fibrosis
Source: J Transl Med. 2019 Aug 23;17:280. doi: 10.1186/s12967-019-2027-8 (PMC6706941; doi:10.1186/s12967-019-2027-8)
Supplement: Supplementary file 1 — Additional file 1. Additional materials and methods. [file 12967_2019_2027_MOESM1_ESM.docx]

***Journal of Translational Medicine 2019***

**Additional file 1**

**A Novel Role of Glutathione S-transferase A3 in inhibiting Hepatic Stellate Cell Activation and Rat Hepatic Fibrosis**

Haihua Chen^1^, Qixin Gan^2^, Congying Yang^3^, Xiongqun Peng^4^, Jiao Qin^5^, Sisi Qiu^6^, Yanzhi Jiang ^1^, Sha Tu^1^, Ying He^1^, Shenglan Li^1^, Huixiang Yang^1^, LijianTao^7^, Yu Peng ^1^

^1^Department of Gastroenterology, Xiangya Hospital, Central South University, 87 Xiangya Road, Changsha, 410008, China

^2^Department of Radiology, Zhuzhou Hospital of Traditional Chinese Medicine,The First Affiliated Hospital of Hunan College of Traditional Chinese Medicine, Zhuzhou, 412000, China

^3^Department of Endoscopic Medical Center, The Affiliated Cancer Hospital of Xiangya School of Medicine Central South University, 283 Tongzipo Road, Changsha, 410013, China

^4^Department of Gastroenterology, Changsha Central Hospital, 161 South Shaoshan Road, Changsha, 410004, China

^5^Department of Nephropathy, Changsha Central Hospital, 161 South Shaoshan Road, Changsha, 410004, China

^6^Department of Ultrasonography, The Third Xiangya HospitalCentral South University, 138 Tongzipo Road, Changsha, 410013, China

^7^Department of Nephropathy, Xiangya Hospital, Central South University, 87 Xiangya Road, Changsha, 410008, China

**Corresponding Athour:**

**Yu Peng, Ph.D.**

Department of Gastroenterology, Xiangya Hospital , Central South University

87 Xiangya Road, Changsha, Hunan 410008, China

mail: [pengyu918@csu.edu.cn;](mailto:pengyu918@csu.edu.cn;)

Tel.: 86 731 84327106; Fax: 86 731 88879602

mail: pengyu918@csu.edu.cn

***Additional materials and methods***

***Histological and immunohistochemical examination***

Liver tissues were fixed in 10% formalin for 24 h and embedded in paraffin. Sections were cut to 4μm thick and stained with hematoxylin and eosin (H&E), Masson’s trichrome or Sirius red. Degree of necroinflammatory liver injury, semiquantitative evaluation of liver fibrosis and the Sirius Red-stained positive area were analyzed as described previously[43, 44]. Expression of 4-HNE in the liver was analyzed by immunohistochemical examination as previously[45]. Briefly, sections (4 µm thick) were dried at 65°C for 1 h followed by dewaxing in xylene for ten minutes. Then, sections were dehydrated in graded alcohol following blockade of endogenous peroxidase activity with 3% hydrogen peroxide in methanol for 20 min at room temperature. Thereafter, sections were washed in phosphate-buffered saline for three times, and then antigen retrieval was performed by incubation in 6.5 mM citrate buffer (pH 6.0) at 100℃ for 10 min. After cooling to room temperature, sections were washed in phosphate-buffered saline three times and then incubated with 5% normal goat serum for 20 min to block nonspecific binding. The slides were incubated with a primary antibody rabbit anti-4-HNE (1/200, #ab46545) abtained from Abcam (Cambridge, MA, USA) overnight at 4°C. After washing with phosphate-buffered saline, secondary antibodies were applied to the sections, and staining was developed by incubating the sections with diaminobenzidine for 15 seconds. After immunostaining, sections were counterstained with hematoxylinand and the 4-HNE-positive areas were measured by Image-Pro Plus 6.0 (Media Cybernetics Inc., MD, USA)[46].

***MDA quantification***

MDA is a byproduct of lipid peroxidation. It was evaluated by Thiobarbituric Acid Reactive Substances (TBARS) Assay kit (R&D Systems, MN, USA) following the manufacturer’s protocol.

***Analysis of mRNA expression***

RNA was extracted with TRIzol reagent (Invitrogen, NY, USA) following manufacturer’s instructions, and cDNA was synthesized using a Reverse Aid First-Strand cDNA synthesis kit (Thermo Fisher Scientific,NY, USA). Quantitative reverse transcription polymerase chain reaction was performed by CFX96 Real-Time System(Bio-Rad, CA, USA) using a SYBR green real-time PCR kit (Thermo). Primers for rat GSTA3 and GAPDH were designed from their GenBank sequences and synthesized by Sangon Biotech (Shanghai, China) (GSTA3 Forward 5’-AACCGTTACTTTCCTGCCTTTG-3’, Reverse5’-GCCCTGCTCAGCCTATTGC-3’; GAPDH Forward 5’-CAGTGCCAGCCTCGTCTCAT -3’, Reverse5’-AGGGGCCATCCACAGTCTTC -3’). The relative expression of GSTA3 was normalized to GAPDH. The expression level (2^−⊿⊿Ct^) of mRNAs were calculated as described previously[47].

***Western blotting analysis***

The liver tissues and cell lysates were subjected to immunoblotting analysis. The NE-PER Nuclear and Cytoplasmic Extraction Reagent Kit(Thermo) was used to abtain cytoplasmic and nuclear extract from HSCs. Protein concentration was calculated by a Thermo Scientific Pierce BCA Protein Assay Kit. 10-30 µg proteins were added to 8% or 10% sodium dodecyl sulfate-polyacrylamide gel under reducing conditions and then transferred the protein onto polyvinylidene difluoride membranes (Millipore, MA, USA). After blocked in TBS-T buffer [10 mM Tris·HCl, 150 mM NaCl, 0.1% (v/v) Tween 20, pH 7.6] containing 5% (w/v) skim milk for 1 h at room temperature, the membranes were incubated with primary antibodies overnight at 4°C. Primary antibodies used were: rabbit anti-p-ERK1/2 (#9101S, 1/1000), rabbit anti-ERK1/2 (#4695, 1/1000), rabbit anti-p-P38 (#9211S, 1/1000), rabbit anti-P38 (#9212, 1/1000), rabbit anti-p-JNK(#9251, 1/1000), rabbit anti-JNK (#9252, 1/1000) , rabbit anti-p-GSK-3β(ser9) (#9323,1/1000) from Cell Signaling Technology ( Danvers, MA, USA); mouse β-catenin(#610153, 1/1000) from BD Biosciences (San Jose, CA); rabbit anti-GSTA3 (#ab180928, 1/1000), mouse anti-FN (#ab6328,1/1000) and mouse anti-Histone H3(ab10799,1/1000) from Abcam; mouse anti-α-SMA (#A5228, 1/5000) and mouse anti-GAPDH (#G9295, 1/10000) from Sigma(St. Louis, MO, USA). Then, membranes were incubated with HRP-conjugated secondary antibodies for 1 h at room temperature. The bands were visualized by BIO-RAD MP5000 (Bio-Rad) using enhanced chemiluminescence (ECL) plus WB detection reagents (Advansta, Menlo Park). The bands were quantified with ImageJ (Glyko, Novato), and the results were expressed as the percentage change in the mean band density compared with the control values.

***Cell culture and treatment***

The immortalized rat and human stellate cell lines CFSC-2G (ATCC) and LX2 were used in this study. HSCs were cultured in Gibco Dulbecco's Modified Eagle Medium (DMEM) supplemented with 10%(v/v) fetal bovine serum, 100 U/ml penicillin, and 100 g/ml streptomycin (Invitrogen) at 37°C in a humidified atmosphere of 5% CO2 and 95% air. AKF-PD treatment was performed after the HSCs were serum starved overnight. To assess the effects of AKF-PD on PDGF-BB-induced expression of GSTA3, α-SMA and FN, serum-starved cells were pretreated with AKF-PD (2mM) for 24h and then treated and subsequently stimulated PDGF-BB(10ng/ml) (Peprotech, #100-14B, NJ, USA) or PDGFRβ inhibitor CP673451(2.5 mM, Cell Signaling Technology) for another 24 h in the presence of AKF-PD before cellular protein extraction. To assess the effects of AKF-PD on PDGF-BB-induced intracellular Wnt/β-catenin siganaling (p-GSK-3β), serum-starved cells were pretreated with AKF-PD (2 mM) for 48h and then incubated with/without PDGF-BB (10 ng/mL) for 15 min in the presence of AKF-PD. Cells were pretreated with the GSK-3β inhibitor XAV-939 (10 mM) (Selleck Chemicals, Texas, USA) for 1 h before PDGF-BB treatment. Each experiment was replicated three times.

***Transient transfection assay***

Hepatic stellate cells were transiently transfected with pcDNA3.1(+)-GSTA3 and pcDNA3.1(+) purchased from Genepharma Technology (Shanghai, China). All plasmids were prepared to be endotoxin free (Qiagen, Valencia, CA). Silencer Select Predesigned siRNA of GSTA3 were purchased from Thermo Fisher Scientific, Inc(human GSTA3 sense 5'-CCGCAGAUGCAAAAGCUUUtt-3', antisense 5'-AAAGCUUUUGCAUCUGCGGga-3'; rat GSTA3 sense 5'-AGAGGAAGCCUUUUGAUGAtt-3', antisense 5'-UCAUCAAAAGGCUUCCUCUgg-3'). Cell were prepared in 6-well plates. When cells were cultured at 70–80% confluence, plasmids (2 µg per well) or siRNA (20 nM) were introduced using Lipofectamine 2000 (Invitrogen) following the manufacturer's instructions. After incubation for 6 h, the medium was replaced with culture medium. To assess the effects of GSTA3 on the expression of α-SMA and FN, cells were then cultured for 48 h before being harvested or stimulated with PDGF-BB(10 ng/ml) for another 24 h after being grown in culture medium for 24 h. To evaluate the effects of GSTA3 on MAPK signaling (p-ERK1/2, p-P38, p-JNK) and phosphorylation of GSK-3β(p-GSK-3β), cells were cultured for 24 h before being harvested or stimulated with 10 ng/ml PDGF-BB for another 15 min after being grown in culture medium for 24 h. GSTA3 overexpression and knockdown were determined by WB analysis. Each experiment was replicated three times.

***Measurement of ROS in HSCs***

ROS in HSCs were assessed with a 2',7'-Dichlorofluorescin diacetate (DCFH-DA) obtained from Sigma as previously reported[48]. The chemiluminescence density was detected with a flow cytometry facility Luminex 200^TM^ (Millipore). The cells (1×10^5^ cells) were plated on 6-well plates in DMEM containing 10% fetal bovine serum for 24 h. To evaluate the effects of AKF-PD on ROS accumulation, serum-starved cells were pretreated with AKF-PD (2 mM) for 24 h and then stimulated with/without PDGF-BB (10 ng/mL) for 30 min in the presence of AKF-PD. To evaluate the effects of GSTA3 on ROS accumulation, the cells were separately incubated with negative control siRNA, rat GSTA3 siRNA or human GSTA3 siRNA and Lipofectamine 2000 complex for 6 h, maintained in culture medium for an additional 48 h, and then encubated with PDGF-BB (10 ng/mL) for 30 min. Cells were pretreated with DCFH-DA (10 µM) and N-acetylcysteine (10 mM) for 1 h before PDGF-BB treatment. Each experiment was replicated three times.

For ROS detected by fluorescence microscopy (Millipore), low light conditions was used. Cells were pretreated with AKF-PD for 24h and N-acetylcysteine for 1 h. Before detected, cells were loaded DCFH-DA for 1 h and stimulated with PDGF-BB for 30min. Phosphate buffer saline was used as loading buffer.

**References**

43. Chevallier M, Guerret S, Chossegros P, Gerard F, Grimaud JA. A histological semiquantitative scoring system for evaluation of hepatic fibrosis in needle liver biopsy specimens: comparison with morphometric studies. Hepatology 1994;20:349-355.

44. Fiorucci S, Antonelli E, Morelli O, Mencarelli A, Casini A, Mello T, Palazzetti B, et al. NCX-1000, a NO-releasing derivative of ursodeoxycholic acid, selectively delivers NO to the liver and protects against development of portal hypertension. Proceedings of the National Academy of Sciences 2001;98:8897-8902.

45. Esterbauer H, Schaur RJ, Zollner H. Chemistry and biochemistry of 4-hydroxynonenal, malonaldehyde and related aldehydes. Free Radical Biology and Medicine 1991;11:81-128.

46. Leung C, Herath CB, Jia Z, Goodwin M, Mak KY, Watt MJ, Forbes JM, et al. Dietary glycotoxins exacerbate progression of experimental fatty liver disease. J Hepatol 2014;60:832-838.

47. Winer J, Jung CKS, Shackel I, Williams PM. Development and Validation of Real-Time Quantitative Reverse Transcriptase–Polymerase Chain Reaction for Monitoring Gene Expression in Cardiac Myocytesin Vitro. Analytical Biochemistry 1999;270:41-49.

48. Kitajima N, Numaga-Tomita T, Watanabe M, Kuroda T, Nishimura A, Miyano K, Yasuda S, et al. TRPC3 positively regulates reactive oxygen species driving maladaptive cardiac remodeling. Sci Rep 2016;6:37001.
