# Supplementary figures and images for: A novel role of glutathione S-transferase A3 in inhibiting hepatic stellate cell activation and rat hepatic fibrosis
Source: J Transl Med. 2019 Aug 23;17:280. doi: 10.1186/s12967-019-2027-8 (PMC6706941; doi:10.1186/s12967-019-2027-8)

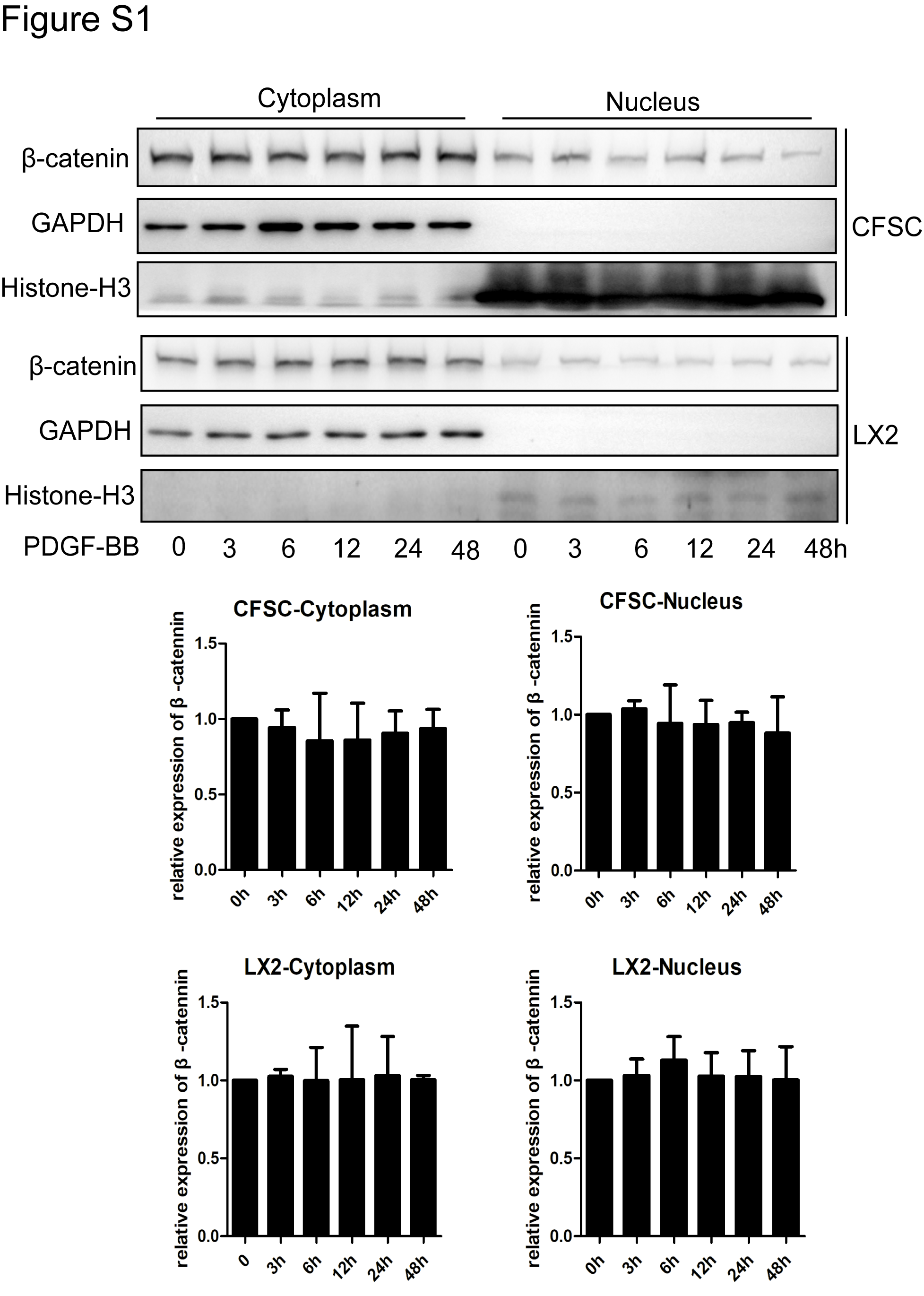

Supplement: Supplementary file 2 — Additional file 2: Figure S1. Expression of β-catenin in cytoplasm and nuclear of HSCs. [file 12967_2019_2027_MOESM2_ESM.tif]
